# King George's Fund for Sailors

**Published:** 1917-11-17

**Authors:** 


### King George's Fund for Sailors.

At a recent meeting of the General Council of King George's Fund for Sailors, held at the Trinity House, the Duke of Connaught said that he felt he might congratulate the Fund upon the cordial reception that had been accorded to it, although he hoped the support already given was nothing to what it would receive in the future. He was confident as to the great advantage that the Fund must be to the marine benevolent institutions, and, through them, to the gallant men of the sea and to their widows and children. Through this Fund the whole country would be able to show its appreciation of the services of the Royal Navy, the Royal Naval Reserve, the Royal Naval Volunteer Reserve, and the men of the Merchant Service. The work of the Fund had been watched with great interest by the King, who, with a desire to mark his sense of its importance and of the benefits which it would confer, had requested him to announce that the King desired to contribute £5,000 towards the admirable work that was being done. This announcement was received with great enthusiasm.

Captain A. W. Clarke (deputy-chairman) said he was pleased to announce that the Fund had now reached £210,000. The expenses in connection with the raising of this amount were only ½ per cent., so that for every £100 subscribed by the public £99 10s. would go direct to the sailors. He thought that this constituted a record in the annals of charity organisation.

Sir Raymond Beck, the chairman of the Finance Committee, then presented a financial report, which was unanimously adopted.

Sir Richard Williams-Bulkeley, in presenting the report of the Collecting Committee, stated that the *Daily Telegraph* appeal continued to render a large measure of material assistance to the King's Fund. The Collecting Committee also wished to acknowledge its gratitude to Lloyd's Exchange and the Commercial Sales Room, Mincing Lane, the Baltic and the Corn Exchange, all of which had contributed generously.

The lead of the Archbishop of Canterbury would no doubt result in a special Sunday being set apart for collections on behalf of the Fund. The report was unanimously adopted.

Sir Thomas Devitt proposed: "That the General Council, in expressing their loyal and humble duty to His Majesty the King, wish also to convey their most grateful thanks for the munificent gift of £5,000 which it has pleased His Majesty to make to the Fund which bears his name. The Council appreciate that in so doing His Majesty once again emphasises his interest in his sailor population and his desire that the nation may liberally and splendidly respond to the appeal which is now being made."

